# *Punica granatum* peel extracts: HPLC fractionation and LC MS analysis to quest compounds having activity against multidrug resistant bacteria

**DOI:** 10.1186/s12906-017-1766-4

**Published:** 2017-05-03

**Authors:** Ilyas Khan, Hazir Rahman, Nasser M. Abd El-Salam, Abdul Tawab, Anwar Hussain, Taj Ali Khan, Usman Ali Khan, Muhammad Qasim, Muhammad Adnan, Azizullah Azizullah, Waheed Murad, Abdullah Jalal, Noor Muhammad, Riaz Ullah

**Affiliations:** 1 Departmen of Microbiology, Kohat University of Science & Technology, Kohat, Pakistan; 2 Departmen of Microbiology, Abdul Wali Khan University, Mardan, Pakistan; 3 Riyadh Community College, King Saud University, Riyadh, 11437 Saudi Arabia; 4 National Institute for Biotechnology and Genetic Engineering, Faisalabad, Pakistan; 5 Department of Botany, Abdul Wali Khan University, Mardan, Pakistan; 6 Department of Botany, Kohat University of Science & Technology, Kohat, Pakistan; 7 Institute of Biotechnology & Genetic Engineering, The University of Agriculture, Peshawar, Pakistan; 8 Department of Biotechnology & Genetic Engineering, Kohat University of Science & Technology, Kohat, Pakistan; 9 Department of Chemistry, Government College Ara Khel, Frontier Region Kohat, Pakistan

**Keywords:** *Puncia granatum* peel extracts, HPLC fractions, Anti-MDR activity, Valoneic acid dilactone, Hexoside, Coumaric acid

## Abstract

**Background:**

Medicinal plants are rich source of traditional herbal medicine around the globe. Most of the plant’s therapeutic properties are due to the presence of secondary bioactive compounds.

**Methods:**

The present study analyzed the High Pressure Liquid Chromatography (HPLC) fractions of *Puncia granatum* (peel) extracts (aqueous, chloroform, ethanol and hexane) against multidrug resistant bacterial pathogens (*Acinetobacter baumannii, Escherichia coli, Pseudomonas aeruginosa and Staphylococcus aureus*). All the fractions having antibacterial activity was processed for bioactive compounds identification using LC MS/MS analysis.

**Results:**

Among total HPLC fractions (*n* = 30), 4 HPLC fractions of *P. granatum* (peel) showed potential activity against MDR pathogens. Fraction 1 (F1) and fraction 4 (F4) collected from aqueous extract showed maximum activity against *P. aeruginosa*. Fraction 2 (F2) of hexane showed antibacterial activity against three pathogens, while ethanol F4 exhibited antibacterial activity against *A. baumannii*. The active fractions were processed for LC MS/MS analysis to identify bioactive compounds. Valoneic acid dilactone (aqueous F1 and F4), Hexoside (ethanol F4) and Coumaric acid (hexane F2) were identified as bioactive compounds in HPLC fractions.

**Conclusion:**

*Puncia granatum* peel extracts HPLC fractions exhibited potential inhibitory activity against MDR bacterial human pathogens. Several bioactive compounds were identified from the HPLC fractions. Further characterization of these compounds may be helpful to conclude it as therapeutic lead molecules against MDR pathogens.

## Background

Medicinal plants are widely used as herbal medicine. More than half of world population in Asia, Latin America and Africa uses medicines from plants origin [1, 2]. Traditional medicines are available in the form of powder, pastes, decoction, infusion and pills for the treatment of various human diseases [3, 4]. Most of the plants are naturally producing a wide verity of bioactive molecules that can be obtained from different parts of the plants and used as antibacterial agents [5, 6].

Recent emergence of drug resistance in bacteria revival search to explore alternative strategy to develop new antibacterial compounds [7]. *Puncia granatum* commonly known as pomegranate, and the peel extract has many beneficial effects including antiviral, immune modulation, diuretic and anthelmintic [8]. According to Dahham et al. *P. granatum* peel extract has both antibacterial and antifungal activity [9].

Antibacterial activity of *P. granatum* peel extracts is already reported but these studies not concluding about the bioactive compounds which have anti-MDR activity. In the present study, gradient HPLC fractions were prepared and only those fractions with antibacterial potential were processed for LC MS/MS analysis to identify bioactive compounds. Findings of the study will be helpful for further elucidation of lead molecules from *P. granatum* for possible therapeutic applications.

## Methods

The current study was undertaken to document the antibacterial activity of *P. granatum* chromatographic fractions against multidrug resistant (MDR) pathogenic bacteria isolates at the Department of Microbiology, Kohat University of Science and Technology (KUST), Kohat.

### Collection and processing of medicinal plants

*P. granatum* (peel extract) plant was collected from Charsadda, Khyber Pakhtunkhwa, Pakistan, and identified at the Herbarium of Botany Department, KUST, where the voucher specimen was deposited under reference number 10051/PG. Plant was subjected to grinding process as described [10]. Briefly *P. granatum* was first washed with tap water, air dried and then chopped into small pieces. After cutting and slicing the collected plant pieces were dried for further processing.

### Preparation of *P. granatum* extracts

Following grinding process, extraction was done as described [11]. Briefly, 1000 g of plant dried powder was soaked in 4 L of all selected solvent (aqueous, chloroform, ethanol and hexane) and incubated it for three weeks. Following filtration, plant’s extracts were then processed in rotary evaporator at 44 °C.

### Gradient HPLC fractionation of *P. granatum* extract

Solidified extracts (hexane ethanol, chloroform and aqueous) of plants were processed for HPLC fractionation by dissolving in 60% methanol as described previously [12]. Briefly, one gram of dried plant powder was dissolved in 10 mL of 60% methanol and centrifuged at 40000 rpm for 5 min. After centrifugation, filtration was done twice for each sample. Before samples subjected to HPLC separation, the column was washed with 60% methanol. Then chromatographic fractionation was done using the PerkinElmer LC technology of C-18 column. This HPLC system configure with Flexar Isocratic LC Pump (N2910400), Flexar solvent Manager (N2600581), 3-cannal Vacuum Degassing and analytical tubing kit (N2910430). HPLC system default pressure was set at 2000 psi, while fractionation was done under 1820–1950 psi pressure and wavelength at 250 nm. About 30 HPLC fractions were multiply collected and analysed.

### Collection of multidrug resistant (MDR) bacteria

Pure cultures of the MDR human pathogens [13] were obtained from the Department of Microbiology, KUST and confirmed on the basis of culture, microscopy and biochemical assays.

### Antibacterial activity of HPLC fractions of *P. granatum*

Overnight fresh cultures (adjusted to standard of 0.5 McFarland turbidity) were inoculated on Muller Hinton agar. In each plate, three HPLC fractions of *P. granatum* plant with negative control (DMSO) were poured in separate well. Antibiotic disc Ceftriaxone (CRO) was incorporated as positive control. All the plates were incubated at 37 °C for 24 h and interpreted the results as per guidelines [14].

### LC MS/MS analysis

The fractions, exhibited antimicrobial activity, were further processed for identification of bioactive compounds by LC MS/MS (LTQ XL, Thermo Electron Corporation, USA) analysis as described earlier [15]. The detection was performed through direct injection mode with Electron Spray Ionization (ESI) probe, at positive-mode. The capillary temperature was kept at 280 °C, while the sample flow rate was set at 8 μL/min. The mass range was selected from 50 to 1000 m/z. The collision induced dissociation energy (CID) during MS/MS was kept in the range of 10 to 45, depending upon the nature of parent molecular ion. As a mobile phase, the ratio of methanol and acetonitrile was 80:20 (*v*/v) for the HPLC fractions of *P. granatum*. The MS parameters for each compound were optimized to ensure the most favorable ionization, ion transfer conditions and attained optimum signal of both the precursor and fragment ions by infusing the analytes and manually turning the parameters. The source parameters were identical for all of the analytes.

### Bioinformatics and data analysis

Chemical structure and other parameters for each compound was searched using online database software (www.chemspider.com). The Xcalibur 2.2 software (Thermo Fisher Scientific, USA) was used for data acquisition and analysis.

## Results

About 30 HPLC fractions were collected from four extracts (hexane ethanol, aqueous and chloroform) and processed for antibacterial activity. All the fractions activity was measured against the known MDR bacterial isolates. Among 30 fractions, only four HPLC fractions exhibited antibacterial activity while the remaining fractions showed no antibacterial activity. Briefly, fraction 1 (F1) of aqueous extract showed inhibitory activity against *P. aeruginosa* (15 ± 0.1 mm) and *E. coli* (13 ± 0.2 mm). Similarly aqueous extract fraction 4 (F4) exhibited against *A. baumannii* (15 ± 0.5 mm) and *P. aeruginosa* (14 ± 0.6 mm).

HPLC fractions (F1, F2, F3, and F4) of hexane extract were subjected for anti-MDR activity against *A. baumannii, P. aeruginosa, S. aureus* and *E. coli*. Only fraction 2 (F2) exhibited activity against *E. coli*, *S. aureus* (11 ± 0.3 mm) and *P. aeruginosa* (10 ± 0 mm). The chloroform fractions showed no antibacterial activity; however, F4 of ethanol extract showed activity against *A. baumannii* (14 ± 0.5) (Table 1).

**Table 1:** Zone of inhibition showed by HPLC fractions of *P. granatum* peel extract

S.No	Bacteria	*Puncia granatum* extracts and their fractions				+ive control (CRO) (mm)	-ive control (DMSO) (mm)
		Aqueous extract		Hexane extract	Ethanol extract		
		F1	F4	F2	F4		
1	*E. coli*	13 ± 0.3	0	12 ± 0.7	0	16 ± 0.3	0
2	*S. aureus*	0	0	11 ± 0.3	0	17 ± 0.8	0
3	*A. baumannii*	0	15 ± 0.5	0	14 ± 0.5	20 ± 0.5	0
4	*P. aeruginosa*	0	14 ± 0.6	10 ± 0	0	15 ± 0.3	0

*F* Fraction, ± standard error of given value, *CRO* Ceftriaxone as positive control, *DMSO* Dimethyl sulfoxide as negative control

**Fig. 1:**
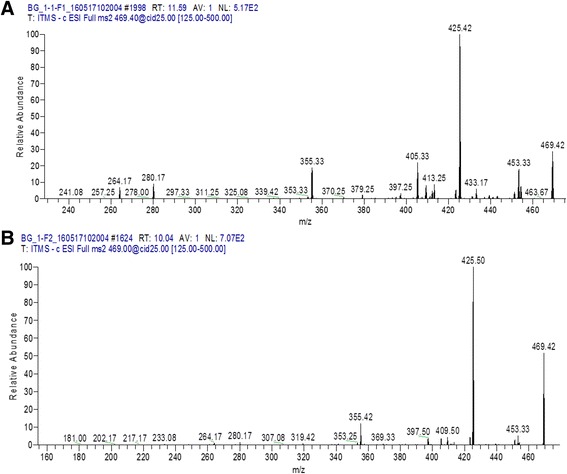
**a** LC MS/MS Chromatogram of fraction 1 (F1), and (**b**) fraction 4 (F4) from aqueous extract of *P. granatum* peel showing Valoneic acid dilactone

**Fig. 2:**
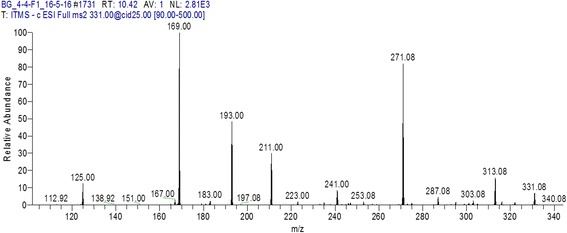
LC MS/MS Chromatogram of fraction 4 (F4) from ethanol extract of *P. granatum* peel showing monogalloyl-hexoside and hexahydroxydiphenoyl-hexoside (HHDP-hexoside)

**Fig. 3:**
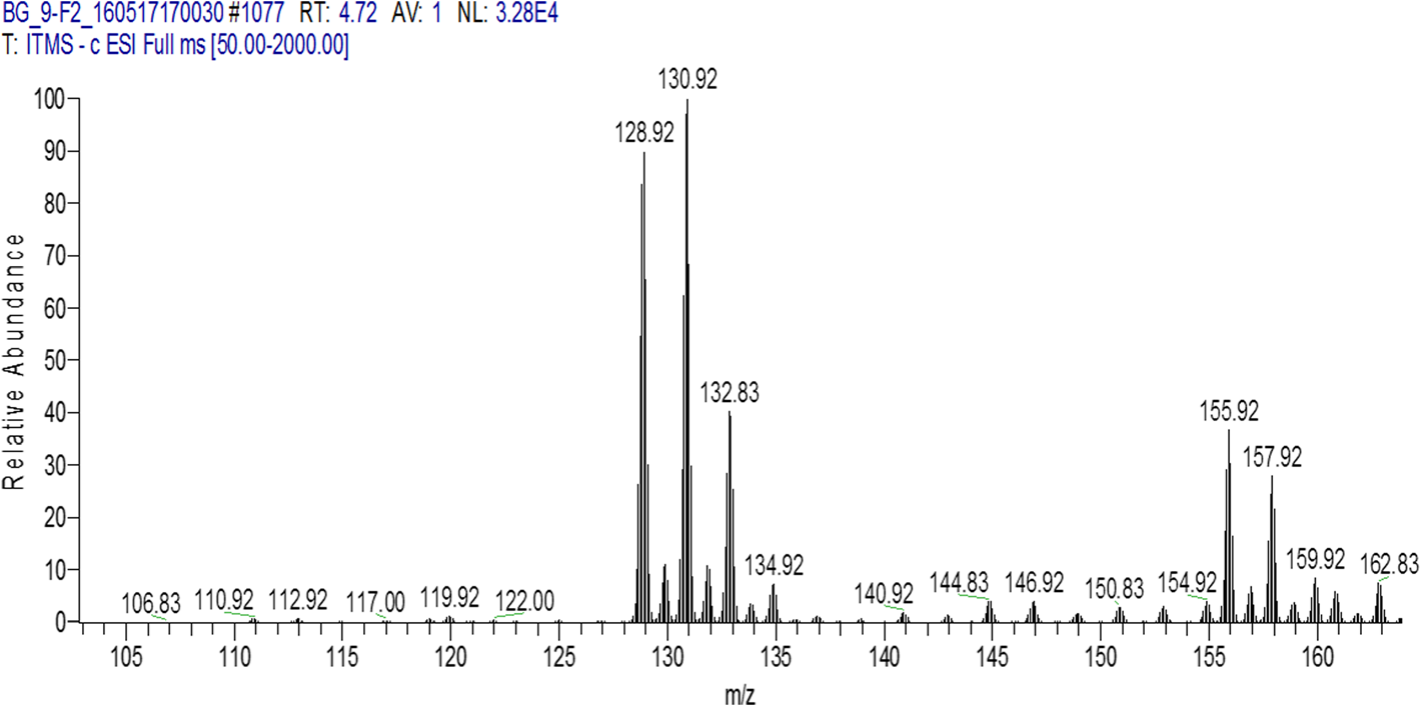
LC MS/MS Chromatogram of fraction 2 (F2) from hexane extract of *P. granatum* peel showing Coumaric acid

Those fractions which have antibacterial activity were processed for identification of bioactive compounds using LC MS/MS analysis. F1 and F4 from aqueous extract of *Puncia granatum* were identified as valoneic acid dilactone. However, F4 from ethanol extract of *P. granatum* plant explored as monogalloyl-hexoside and hexahydroxydiphenoyl-hexoside (HHDP-hexoside). Similarly F2 of hexane extract have the presence of coumaric acid (Figs. 1, 2, 3). All the chemical characteristics of identified compounds are listed in Table 2.

**Table 2:** Chemical characteristics of identified compounds from *P. granatum* peel by LC MS/MS

S.No	Fraction source	Compound	Molecular formula	Average mass (g/mol)	Structure
1	Aqueous	Valoneic acid dilactone	C_21_H_10_	470.296	
2	Ethanol	Monogalloyl-hexoside	C_13_H_16_O_10_	332.260	
3	Ethanol	Hexahydroxydiphenoyl-hexoside	C_20_H_18_O_14_	482.349	
4	Hexane	Coumaric acid	C_9_H_8_O_3_	164.158	

## Discussion

Processing a single plant in different formulations may show excellent results against different pathogens and can be used to cure a wide range of diseases. However, medicinal plant has various bioactive components, so they are needed to be separated on HPLC and then tested for antimicrobial activity [16]. Previously no data is available on the HPLC fractionation of *P. granatum* but many previous studies evaluated the antimicrobial potential of *P. granatum* extracts.

HPLC fractions of *P. granatum* plant were evaluated for activity against MDR human bacteria pathogens. It was observed that aqueous extract of HPLC F1 and F4 were effective against *E. coli*, *A. baumannii* and *P. aeruginosa*. This reflects solubility of valuable compounds of *P. granatum* in aqueous extract.

Chromatographic fractions (F1, F2, F3, and F4) of hexane extract showed anti-MDR activity against *A. baumannii, P. aeruginosa, S. aureus* and *E. coli*. Among these fractions, F2 exhibited maximum activity against *S. aureus* (Gram positive) and *P. aeruginosa* (Gram negative), while F4 of ethanol was only effective against *A. baumannii*. In a previous study bioactive compounds of *P. granatum* were extracted using aqueous, methanol, ethanol, acetone, ether and chloroform solvents. However, in a study the water soluble alkaline fractions contain the highest number of phenolic acids and they were most effective against Gram positive bacteria [17]. Martinsa et al. also revealed that the antibacterial activity of fractions from *Larrea tridentata* were more effective by inhibiting the growth of Gram-positive bacteria when compare with Gram-negative bacteria [18].

HPLC fractions which were positive for antibacterial activity were further processed for LC MS/MS analysis for compound identification. It was found that aqueous extract by gradient HPLC, F1 and F4, were identified as Valoneic acid dilactone by means of LC MS/MS appeared to be more effective against Gram negative MDR bacteria. Valoneic acid dilactone has antimicrobial potential and is already reported in methanolic extracts of *Lagerstroemia speciosa* leaves [19].

When F2 fraction of hexane extract was evaluated, a compound named Coumaric acid was identified by LC MS/MS analysis. Coumaric acid was isolated from *Securigera securidaca* and showed bioactivity [20]. F4 from ethanol extract of *P. granatum* plant explored as Monogalloyl-hexoside and hexahydroxydiphenoyl-hexoside (HHDP-hexoside) and these compounds showed potential activity against selected bacteria. Katarina et al. have reported hexoside as potential antibacterial compound from methanolic extracts of *Cotinus coggygria Scop*. leaves [21].

## Conclusion

HPLC fractions of *Puncia granatum* peel extracts showed inhibitory activity against the clinical MDR isolates. The present study has for the first time identified bioactive compounds from HPLC fractions of *P. granatum*. Further elucidation of these compounds may be helpful to search new alternative biotherapeutics against MDR pathogens.
